# Molecular investigation of the 7.2 kb RNA of murine cytomegalovirus

**DOI:** 10.1186/1743-422X-10-348

**Published:** 2013-12-02

**Authors:** Toni M Schwarz, Lysa-Anne M Volpe, Christopher G Abraham, Caroline A Kulesza

**Affiliations:** 1 Department of Microbiology, University of Colorado School of Medicine, MS8333, 12800 E. 19th Ave, Aurora, Colorado 80045, USA

**Keywords:** Cytomegalovirus, Non-coding RNA, Persistence

## Abstract

**Background:**

HCMV encodes a stable 5 kb RNA of unknown function that is conserved across cytomegalovirus species. *In vivo* studies of the MCMV orthologue, a 7.2 kb RNA, demonstrated that viruses that do not express the RNA fail to establish efficient persistent replication in the salivary glands of mice. To gain further insight into the function and properties of this conserved locus, we characterized the MCMV intron in finer detail.

**Methods:**

We performed multiple analyses to evaluate transcript expression kinetics, identify transcript termini and promoter elements. The half-lives of intron locus RNAs were quantified by measuring RNA levels following actinomycin D treatment in a qRT-PCR-based assay. We also constructed a series of recombinant viruses to evaluate protein coding potential in the locus and test the role of putative promoter elements. These recombinant viruses were tested in both *in vitro* and *in vivo* assays.

**Results:**

We show that the 7.2 kb RNA is expressed with late kinetics during productive infection of mouse fibroblasts. The termini of the precursor RNA that is processed to produce the intron were identified and we demonstrate that the m106 open reading frame, which resides on the spliced mRNA derived from precursor processing, can be translated during infection. Mapping the 5′ end of the primary transcript revealed minimal promoter elements located upstream that contribute to transcript expression. Analysis of recombinant viruses with deletions in the putative promoter elements, however, revealed these elements exert only minor effects on intron expression and viral persistence *in vivo*. Low transcriptional output by the putative promoter element(s) is compensated by the long half-life of the 7.2 kb RNA of approximately 28.8 hours. Detailed analysis of viral spread prior to the establishment of persistence also showed that the intron is not likely required for efficient spread to the salivary gland, but rather enhances persistent replication in this tissue site.

**Conclusions:**

This data provides a comprehensive transcriptional analysis of the MCMV 7.2 kb intron locus. Our studies indicate that the 7.2 kb RNA is an extremely long-lived RNA, a feature which is likely to be important in its role promoting viral persistence in the salivary gland.

## Background

Human cytomegalovirus (HCMV) is a member of the β-herpesvirus subfamily and a widespread human pathogen [1,2]. HCMV infections cause life-threatening illness in the immunocompromised, including bone marrow and solid organ transplant recipients, AIDS patients and patients undergoing cancer chemotherapy. In addition, HCMV infection of the fetus is the leading cause of virally induced birth defects, typically presenting as hearing loss and developmental deficits that impact over 40,000 newborns annually in the US [3-5].

HCMV infection of healthy, immunocompetent individuals elicits a robust host immune response that effectively limits virus replication and pathogenesis. Despite this immune response, HCMV has adapted to long-term infection of humans by balancing persistent replication with clearance by the immune response. HCMV persistently replicates in glandular epithelial tissue and eventually establishes a life-long latent infection of the host. Glandular epithelial cells, such as those in the salivary gland, are key sites of HCMV persistent replication *in vivo,* and contribute significantly to viral transmission. Healthy individuals may secrete virus in saliva, breast milk, and urine for long periods of time following primary infection [2,3]. In order for HCMV to successfully persist, it has evolved to replicate in cell types where the full replication cycle elicits little to no cytopathic effect, such as glandular epithelial cells and some types of endothelial cells [6-8]. In addition, the ability to persistently replicate in the host likely depends on reduced immune recognition of virus-infected cells at these specialized sites [9,10]. Few viral determinants that mediate cytomegalovirus persistence have been identified and little is known about the specific molecular functions that facilitate persistence. These include virus-encoded micro-RNAs and a conserved, virus-encoded G-protein-coupled receptor [11-13]. In addition, we previously identified a long, non-coding RNA (lncRNA), expressed by all cytomegaloviruses, that we showed to also be an important viral determinant of persistence [14].

During lytic replication, HCMV expresses a 5 kb lncRNA of unknown function (also referred to as RNA5.0) [14-16]. We showed that this RNA is dispensable for replication in cultured cells and is a stable intron produced by the processing of a large precursor transcript expressed from the genomic region flanked by UL105 and UL111A. Orthologous loci are present in every β-herpesvirus genome examined thusfar, although there is little conservation of sequence or RNA size between different CMV species. Each locus shares some common features, including a high AT sequence content (~60%), and the presence of many homopolymeric stretches of A or T residues. The consensus splice donor sequence that defines the 5′ end of the RNA produced from each locus is also well conserved.

Cytomegaloviruses exhibit strict species specificity and there is no animal model for HCMV infection. Murine cytomegalovirus (MCMV) infection of the mouse is widely used as an outstanding small-animal model of HCMV infection for several reasons. HCMV and MCMV share similar genomic sequence and organization and undergo similar replication cycles [17]. Like HCMV, MCMV acutely infects multiple tissues in the mouse, persistently replicates in the salivary gland and establishes a lifelong latent infection of the host [18]. Therefore, MCMV infection of the mouse is an excellent surrogate for the study of pathogenesis *in vivo*. MCMV expresses a 7.2 kb ortholog of the HCMV 5 kb RNA [19]. We have shown that recombinant MCMV that does not express the 7.2 kb RNA replicates normally in cultured fibroblasts, but is unable to progress from the acute to the persistent phase of infection in mice. We identified a short hairpin sequence near the 3′ end of the intron that is required for accumulation of the RNA during infection. Persistent replication in the salivary gland of the mouse depends on the accurate processing and stable retention of the intron, since recombinants with a mutation in the splice donor site or deletion of the hairpin sequence fail to transition to the persistent phase of replication *in vivo*. The specific molecular function of this conserved RNA is unknown, but we hypothesize that it mediates processes essential for the virus to evade immune surveillance and/or replicate efficiently at sites of viral persistence.

To gain further insight into the function and properties of this conserved locus, we characterized the MCMV intron in finer detail. We confirmed that the intron locus RNAs are expressed with late gene kinetics. The 7.2 kb intron has an unusually long half-life, whereas the spliced mRNA that results from processing of the intron is metabolized with kinetics similar to most cellular mRNAs. Investigation of the promoter sequence that controls expression of the intron locus revealed a minimal promoter sequence contributing to the transcriptional output of the locus. Although there is no evidence for translation of the intron itself, we discovered that the spliced mRNA encodes a small protein that co-localizes with the RNA within the nuclei of infected cells. Importantly, we show that the RNA is not required for trafficking of virus to the salivary gland *in vivo,* supporting our hypothesis that the 7.2 kb RNA functions to either evade the host response or maintain viral replication at sites of persistence.

## Results

### The MCMV 7.2 kb intron locus is transcribed with true late kinetics

**Figure 1:**
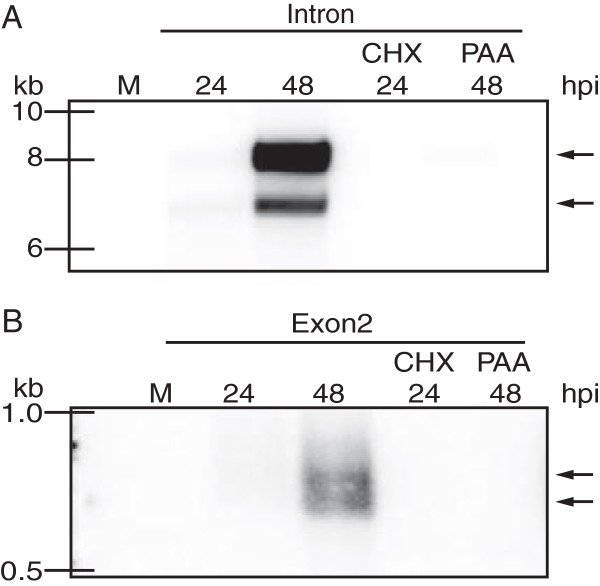
**Expression kinetics of intron locus transcripts.** Total RNA was harvested from infected mouse fibroblasts (MOI = 1.0) at the indicated times and analyzed by northern blot analysis using radio-labeled, antisense RNA probes specific for **(A)** the intron or **(B)** exon 2 of the spliced mRNA. To determine the kinetics of intron locus expression, cells were pre-treated with either PAA or CHX for 1 hour prior to infection. Total RNA was prepared at the indicated times.

To determine the transcription kinetics of the 7.2 kb intron locus during productive MCMV infection, northern blot analysis was performed on total RNA prepared from cells treated with the translation inhibitor cycloheximide (CHX) or the DNA replication inhibitor phosphonoacetic acid (PAA) prior to MCMV infection. CHX pre-treatment of cells inhibits translation of immediate early (IE) genes blocking subsequent transcription of both early and late classes of viral genes. PAA treatment blocks DNA replication, on which expression of late (L) genes is dependent [1,2]. Transcription of the intron (Figure 1A) and the spliced mRNA (Figure 1B) was inhibited by both CHX and PAA treatment. This data indicates that the intron locus RNAs are transcribed with the late class of viral genes during productive infection in fibroblasts.

In high resolution northern blot analyses specific for the intron RNA, we routinely observed a doublet of bands near 7.2 kb: a major species at approximately 8.0 kb and a minor species migrating faster at 7.2 kb (Figure 1A). These observations were made with multiple intron-specific probes (data not shown). We have been unable to ascertain the basis for this difference in size, although we hypothesize it may be due to effects of lariat secondary structure on RNA migration during electrophoresis resulting in slower migration (data not shown). Likewise, we also observed a doublet of closely migrating bands in northern blot analyses of the spliced RNA product of the locus (Figure 1B). We cannot account for the difference in size based on sequencing of the 5′ and 3′ Rapid Amplification of cDNA Ends (RACE) products (see below). It is possible that we did not capture both species in the RACE reactions but we think it is likely the differences in size reflect variability in 3′ end processing and poly-adenylation that we cannot assess (see Discussion).

### Location of transcriptional start sites and RNA processing signals

**Figure 2:**
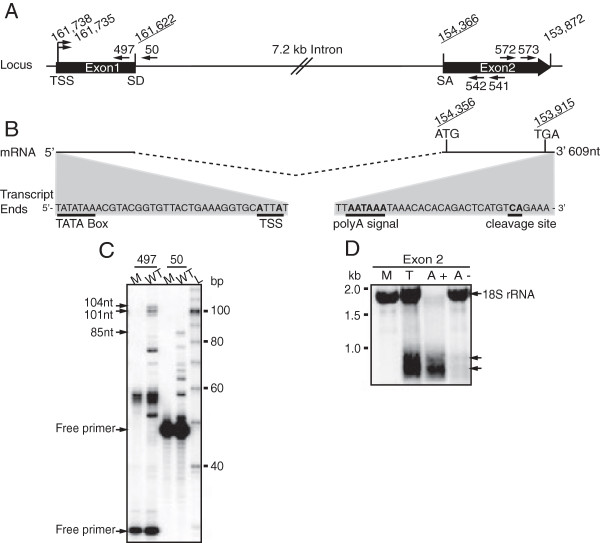
**Mapping of the intron locus. (A)** Diagram of the genomic region encompassing the primary transcript of the 7.2 kb intron, illustrating the transcriptional start sites (TSS) and the splice donor (SD) and splice acceptor sites (SA). The location of primers used for primer extension or RLM-RACE are indicated. **(B)** Diagram of the spliced mRNA and the 5′ and 3′ ends that were identified by RLM-RACE using total RNA harvested from infected mouse fibroblasts at 48 hours post infection (hpi). Putative TATA box directly upstream of the transcriptional start sites is indicated. Also shown are the start and stop codons of the m106 ORF encoded on the second exon of the mRNA as well as the poly-adenylation and cleavage sites used in processing this mRNA. **(C)** Primer extension analysis was performed on total RNA harvested from either mock-infected (M) or MCMV (WT) infected mouse fibroblasts at 48 hpi. Radiolabeled primers were used to validate the 5′RLM-RACE products (primer 497) and confirm the known 7.2 kb splice donor site (primer 50) as a control. **(D)** Northern blot analysis of total RNA (T) harvested from infected mouse fibroblasts and fractionated for either polyadenylated (A+) or non-polyadenylated (A-) RNA. The blot was hybridized with a radiolabeled, antisense RNA probe specific for exon 2.

While the splice donor and acceptor sites used in the processing of the 7.2 kb RNA from the primary transcript were previously mapped, the termini of the primary transcript were not determined [19]. To identify the 5′ and 3′ ends of the primary transcript, we cloned and sequenced PCR products generated by RACE (summarized in Figure 2A-B). To identify the 5′ end of the precursor RNA and capture the predicted intron-exon junctions in the 5′ RACE reaction, we used nested PCR primers specific for the predicted second exon located 3′ of the intron (primers 541 and 542 Table 1, Figure 2A). Sequencing of cloned RACE products identified two transcriptional start sites located three nucleotides apart at positions 161,738 and 161,735 in the MCMV genome (sequence coordinates based on the MCMV Smith strain, Genbank accession #NC004065). Sequence alignment of the 5′-RACE products to MCMV genomic sequence also confirmed the splice donor (SD) and splice acceptor (SA) sequences at nucleotide positions 161,622 and 154,366, respectively, as previously annotated [19]. We also performed primer extension analysis to confirm the transcriptional start sites identified by 5′-RACE. We observed two primer extension products of 101 and 104 nucleotides in length, consistent with the location of the 5′ ends of the spliced RNA as defined by RACE (primer 497 Table 1, Figure 2C).

**Table 1:** Primer sequences

**Target**	**Purpose**	**Sequence 5′ to 3′**
7.2 kb Intron (a and a’)	qPCR	Fwd: GAGTCAGTTCTAACCCATCACG
		Rev: AGCTCGAAAGTTGAACGGG
		Probe: ACGAACGGGTAAAACGGGTAAGGG
Exon2 (b and b’)	qPCR	Fwd: CCACTACCTCTCGATGACAAC
		Rev: AGCGAATTCTAGCGTTACCG
		Probe: CGGAGCCTGCGACTTGTCTGC
Spliced mRNA (c and c’)	qPCR	Fwd: TTATCACACCTGAGCGAACG
		Rev: GCAGAGTTCGATGTGTCCG
		Probe: AGGATGCGAGATGGCGACGG
M54	qPCR	Fwd: AACATATCCCTGCCGATCTTG
		Rev: CAACGCTTTCTACGGTTTCAC
		Probe: ATGCTCCCGTGTCTCCCCATC
GAPDH	qPCR	Fwd: GTGGAGTCATACTGGAACATGTAG
		Rev: AATGGTGAAGGTCGGTGTG
		Probe: TGCAAATGGCAGCCCTGGTG
Actin B	qPCR	Fwd: CTTGATCTTCATGGTGCTAGGAG
		Rev: CGTTGACATCCGTAAAGACCT
		Probe: ACCATGTACCCAGGCATTGCTGA
18 srRNA	qPCR	Fwd: GTTGATTAAGTCCCTGCCCTT
		Rev: ATAGTCAAGTTCGACCGTCTTC
		Probe: ACCGATTGGATGGTTTAGTGAGGCC
Exon1	Primer extension	497:GGCCTTCGGGACGCCGTCACCTCCGCCGCCGC
Exon2	3′RACE	541: GATCGTTGTCGTCTCTGTCGTGTT
	3′RACE	542: TGTCATCGAGAGGTAGTGGAGGAT
	5′RACE	571: ATCCTCCACTACCTCTCGATGACA
	5′RACE	572: AACACGACAGAGACGACAACGATC
	Northern	253: GTCGACATGGCGACGGCGAGCCAGCAA
	Blot	263: GCGGCCGCGTCTACCACCTCGACCACGATT
	Probe	
7.2 kb Intron	Northern	262: CTCCAATCGGCCTAGGAATCCTGGCTAGGT
	Blot	263: AGCAACACGATGCTCTGTGTCGTCGGTCGG
	Probe	
M112/113	pGL3 Construct	459: ACGAAGGTCTTTTCACCGGT
		435: ACCATCTGCTAGGCGGGTCC
7.2PR1	pGL3 Construct	28: AGATAGCGCGGCGTCCGTCG
		349: CTGAGAGCTCCGGGCCTTCGG
7.2PR2	pGL3 Construct	133: AAAAGAAAGTCCGTGACCGGGTCG
		349: CTGAGAGCTCCGGGCCTTCGG

A single 3′ end was identified at nucleotide position 153,872 by sequencing of 3′-RACE products (Figure 2A). This end is located downstream of a putative polyadenylation signal at position 153,898 (Figure 2B). We also examined the polyadenylation status of the spliced RNA by northern blot analysis of oligo(dT)-selected RNA prepared from MCMV-infected cells. The majority of the spliced mRNA from the intron locus was detected in the poly A + fraction of RNA (Figure 2D). 18S rRNA can only be detected in the non-polyadenylated fraction demonstrating that our fractionation protocol efficiently captured polyadenylated mRNA only (Figure 2D lanes A^+^ and A^-^). Taken together, our data suggest that a large precursor RNA is transcribed from the intron locus at late times of infection and processed to yield a single 7.2 kb stable intron and a spliced poly-adenylated mRNA consisting of two exons.

### The m106 open reading frame on the spliced mRNA is translated during infection

**Figure 3:**
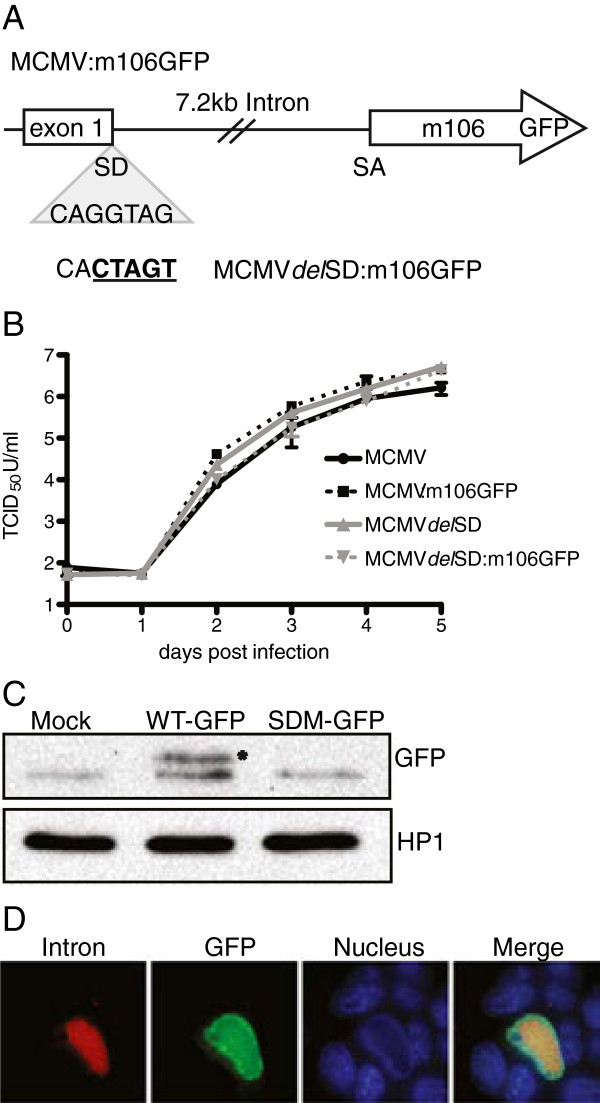
**The m106 open reading frame is translated during MCMV infection. (A)** Diagram of the GFP cassette insertion within the MCMV Smith BAC clone. **(B)** Analysis of recombinant virus replication in mouse fibroblasts. Cells were infected at a multiplicity of 0.05 PFU/cell and viral supernatants were collected daily and titrated. Graph represents two biological replicates. **(C)** Western blot analysis of protein lysates prepared from mock-, MCMV:m106GFP (WT-GFP), or MCMV*del*SD:m106GFP (SDM-GFP) infected cells at 48 h p.i. m106-GFP expression was detected using a polyclonal anti-GFP antibody. Asterisk denotes m106-GFP. **(D)** Mouse fibroblasts were infected with MCMV:m106-GFP (MOI = 0.05). Cells were fixed at 72 h p.i. and m106-GFP protein expression and 7.2 kb intron production was detected by by combined immunofluorescence assay and FISH.

The second exon of the spliced mRNA processed from the primary transcript that produces the 7.2 kb RNA spans a previously annotated open reading frame (ORF) called m106 [17]. Positional orthologues of m106 have been identified in all cytomegaloviruses, including the UL106 ORF of HCMV, yet there is little sequence homology among the group [20-23]. In general, UL106 orthologues score poorly with algorithms designed to predict the potential of an ORF to encode a protein [24,25]. To determine if m106 protein is translated during MCMV replication we constructed two recombinant viruses engineered to express m106 as a GFP fusion at the carboxy-terminus (Figure 3A). The first recombinant virus expresses the m106-GFP fusion from the wild-type MCMV genome (MCMV:m106GFP). The second recombinant virus expressing the m106-GFP fusion also contains a five-nucleotide substitution at the splice donor site that defines the 5′ end of the 7.2 kb RNA (MCMV*del*SD:m106GFP) [19]. This substitution prevents processing of the intron from the primary transcript and we predicted that it would also prevent translation of m106-GFP protein. Both recombinant viruses replicate with wild-type kinetics in multi-step growth analysis in mouse fibroblasts (Figure 3B). Immunoblotting for the m106-GFP fusion protein with antibody specific for GFP only detected protein expression during MCMV:m106GFP infection and not MCMV*del*SD:m106GFP infection, indicating that splicing of the mRNA is necessary for translation of m106 (Figure 3C). Furthermore, this data indicates that cryptic transcriptional initiation does not appear to occur within the unspliced transcript produced by MCMV*del*SD:m106GFP at any significant level.

### m106 and the 7.2 kb intron localize to the nucleus of infected fibroblasts

Although we previously demonstrated in fractionation studies that the HCMV 5 kb intron localizes to the nuclear compartment of infected cells, the specific sub-nuclear localization of the RNA was not examined [14]. Fluorescent in situ hybridization (FISH) was used to visualize the 7.2 kb intron in infected mouse fibroblasts. The FISH staining revealed an even, granular distribution of the 7.2 kb intron throughout the nuclear compartment of infected fibroblasts (Figure 3D). Co-staining for the 7.2 kb RNA and m106-GFP revealed that the RNA and m106 protein are found co-localizing in the nucleus late during infection (Figure 3D). We also observed some m106-GFP protein was localized to the cytoplasm of infected cells.

### The MCMV 7.2 kb intron is highly stable

**Figure 4:**
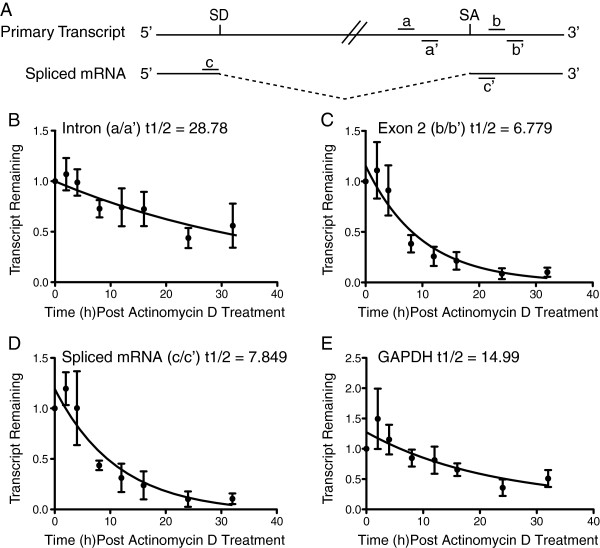
**Half-life analysis of the intron locus transcripts.** Mouse fibroblasts were infected with MCMV (MOI = 1.0). At 30 hours post infection, cells were treated with 4 ug/ml of Actinomycin D. Total RNA was harvested over the indicated time course. Intron, mRNA, and 18 srRNA transcript levels were quantified by qRT-PCR. Intron and spliced mRNA transcripts were normalized to 18 srRNA. The relative quantitative values at time zero hours were adjusted to 100% and transcript remaining was compared relative to time zero. The fitted curve was modeled by one phase decay using a non-linear regression analysis on four biological replicates for each time point. The half-life (t1/2) shown for each transcript is the best-fit value. Bars represent the mean and error bars represent the standard error of the mean (SEM). **(A)** Schematic representation of primer probe sets used for qRT-PCR analysis. SD = splice donor sequence, SA = splice acceptor sequence. Half-life decay curves for the **(B)** 7.2 kb intron using primer probe set a/a’, **(C)** the second exon of the mRNA (b/b’), **(D)** the spliced mRNA using primer probe set c/c’, and **(E)** GAPDH.

The 7.2 kb RNA accumulates to high levels during infection as detected by northern blot analysis, suggesting it is unusually stable for an intron [14,19,26,27]. To quantify transcript stability, we measured RNA decay rates of intron-locus transcripts during MCMV infection. RNA half-lives were quantified by measuring RNA abundance by quantitative RT-PCR at several time points after treatment of infected cells with Actinomycin D. This compound inhibits RNA Polymerase II by intercalating between GC residues thereby blocking processivity of the enzyme. Actinomycin D treatment effectively arrests transcription of RNA pol II-dependent RNAs and allows us to measure relative decay rates. Using this strategy, we calculated the half-life of the 7.2 kb intron to be ~28.8 hours (Figure 4B). In general, the half-life of low-stability RNAs is typically less than 2 hours whereas long-lived RNAs with high stability possess a half-life greater than 12 hours [28]. The half-life of the spliced mRNA derived from processing of the intron was measured using two different primer probe sets: b/b’ targets the second exon and c/c’ spans the splice junction (Table 1, Figure 4A). We measured a half-life of ~6.8 hours using the primer-probe set targeting the second exon (Figure 4C), while a half-life of ~7.8 hours was measured using the primer-probe set that spans the splice junction (Figure 4D). This difference may be accounted for by differences in primer-probe efficiency, processing of the primary transcript, or location of the primer-probe sets in relation to protective secondary structures within the mRNA from degradation machinery. Both half-lives, however, are consistent with the average half-life for a protein coding RNA. As a control, we determined the half-life of GAPDH mRNA to be ~15 hours, similar to published estimates (Figure 4E) [28]. Our data demonstrates that the MCMV 7.2 kb intron is, in fact, unusually stable for an intron RNA.

### Analysis of minimal promoter sequences

**Figure 5:**
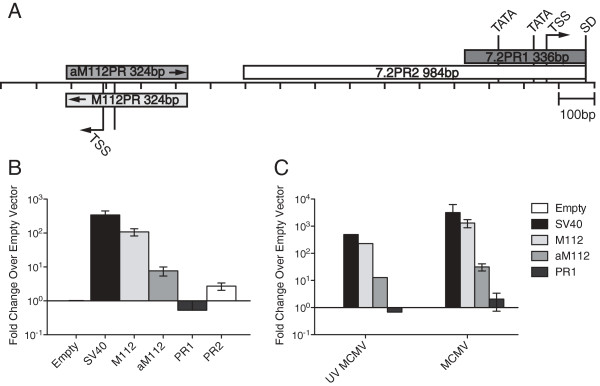
**Analysis of transcriptional activity of putative intron locus promoter elements. (A)** Diagram of genomic location of the viral sequences cloned into pGL3-reporter plasmids. **(B)** pGL3-reporter constructs were co-transfected into mouse fibroblasts and luciferase induction was assayed 24 h p.i. following either mock co-infection, **(C)** UV inactivated co-infection, or MCMV co-infection (MOI = 0.05).

Little data is available regarding the sequence elements driving late transcriptional units of cytomegaloviruses. Therefore, the DNA sequence within the vicinity of the TSSs of the intron locus was examined for transcriptional regulatory sequences. A putative TATA box element was identified 24 nucleotides upstream of the transcriptional start site, as well as an additional TATA box located 127 nucleotides upstream (Figure 2B, Figure 5A). In order to examine the transcriptional activity of these putative minimal promoter elements and the nucleotide sequence surrounding them, DNA sequences from the region between the intron splice donor site and the M112/113 locus were cloned into a luciferase reporter plasmid to quantify transcriptional promoter activity (7.2PR1 and 7.2PR2). The M112/113 locus is located upstream of the intron locus and on the opposite strand. The transcriptional start site and promoter elements controlling the M112/113 locus have been defined [29,30]. Therefore, the M112/113 early-late promoter was cloned into the reporter vector in both sense and antisense orientations (M112PR and aM112PR). To serve as a control, only the M112/113 promoter sequence in the sense orientation induced luciferase activity similar to the control (Figure 5A). Surprisingly, no appreciable induction of luciferase activity was observed for 7.2PR1 or 7.2PR2 relative to the promoterless control vector (Figure 5B). We also found that MCMV co-infection of reporter-tranfected cells did not induce luciferase activity from the minimal 7.2PR1 promoter construct (Figure 5C).

**Figure 6:**
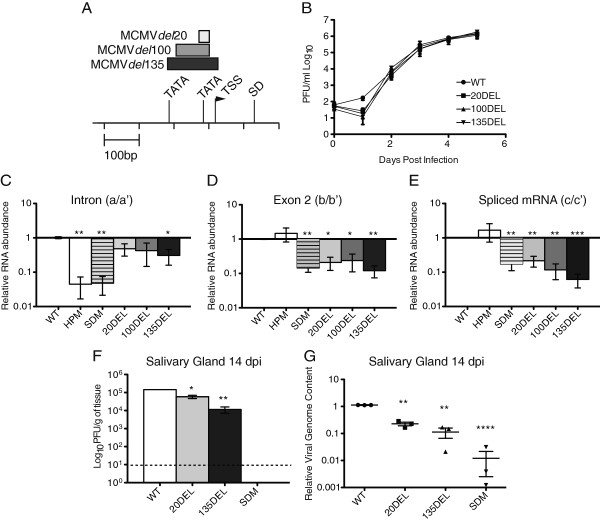
**Deletion mutations in putative viral promoter elements reveal reduction in transcriptional output in cell culture and decreased recovery of infectious virus *in vivo*. (A)** Diagram of the genomic location of intron locus promoter region deletions. **(B)** Multi-step growth analysis of replication of all three recombinant viruses in comparison to WT MCMV. Mouse fibroblasts were infected (MOI = 0.05) and culture supernatants were collected every 24 hours and titrated by plaque assay. Graph represents three biological replicates. **(C**, **D**, **and E)** Quantification of intron locus RNAs in cells infected with promoter mutant viruses. Mouse fibroblasts were infected (MOI = 1.0) and total RNA was harvested 48 hours post infection. Intron locus transcript levels are quantified relative to WT MCMV transcript levels by qRT-PCR. The primer probes sets used are the same as shown in Figure 4A. Graphs represent three biological replicates. **(F and G)** Three-month-old female BALB/c mice were infected with an i.p. dose of 5 x 10^5^ PFU with the indicated viruses. At 14 days post infection, animals were euthanized and tissues collected for analysis of infectious virus yield **(F)** and viral genome number **(G)**. **(F)** Salivary gland homogenates were analyzed by plaque assay on mouse fibroblasts. *p* values represent the Student’s T Test result between WT MCMV infected cells or mice and cells or mice infected with the given recombinant viral mutant for each transcript analyzed (*p < 0.05 **p < 0.01 ***p < 0.001 ****p < 0.0001). WT MCMV = WT; MCMV*del*20 = 20DEL; MCMV*del*100 = 100DEL; MCMV*del135 =* 135DEL*;* MCMV*del*HP = HPM; MCMV*del*SD = SDM.

To examine the contribution of the putative minimal promoter sequences to transcription of the intron locus RNAs in the context of virus infection, three recombinant viruses were made with deletions in this region (Figure 6A). We constructed recombinants with (1) a 20 bp deletion spanning the TSS-proximal TATA box, (2) a 100 bp deletion including the proximal TATA box and upstream sequence, and (3) a 135 bp deletion spanning both the proximal and distal TATA boxes in the putative minimal promoter sequence. All three recombinant viruses replicated similar to wild-type MCMV in multi-step growth analysis (Figure 6B). We quantified RNA expression in cells infected with our panel of recombinant viruses, including previously characterized recombinants with mutations that result in a failure to express significant levels of the intron (MCMV*del*HP and MCMV*del*SD) [19]. The MCMV*del*HP contains a 28 bp deletion spanning a predicted stem loop structure at the 3′end of the intron that is hypothesized to confer stability. Without this stem loop structure, processing of the primary transcript still occurs since the mRNA is detected by qRT-PCR and northern blot analysis, but accumulation of the intron is significantly reduced (Figure 6C). While the splice donor site mutation impacts processing of the precursor transcript and is not expected to affect transcriptional output of the promoter, we predicted that MCMV*del*135 would reduce overall transcript production from the locus. Measured reductions in levels of the intron were only significant for cells infected with the MCMV*del*135 mutant in addition to the MCMV*del*SD and MCMV*del*HP recombinant viruses (Figure 6C). In cells infected with MCMV*del*135, intron abundance was reduced approximately 5-fold while levels of the mature mRNA transcript were reduced by 10-fold (Figure 6C,D, and E). Despite different predicted consequences of the mutations, the reduction of the spliced mRNA transcript abundance is similar between the MCMV*del*SD and the MCMV*del*135.

To determine if reductions in intron and mRNA expression have an effect on the establishment of persistence *in vivo*, mice were inoculated with a subset of our panel of recombinant viruses and viral yields measured in the salivary gland at 14 days post-infection (Figure 6F). We observed a slight reduction in viral yield in the salivary glands of mice infected with MCMV*del*20 and a ten-fold reduction of viral yield in mice infected with MCMV*del*135. Viral genome quantification corroborated the measure of infectious virus within the salivary gland (Figure 6G). However, despite 5–10 fold reductions of intron and mRNA production, neither promoter deletion mutant fully attenuated persistent replication to the levels observed in mice infected with MCMV*del*SD.

### Intron locus products do not influence dissemination to the salivary gland

**Figure 7:**
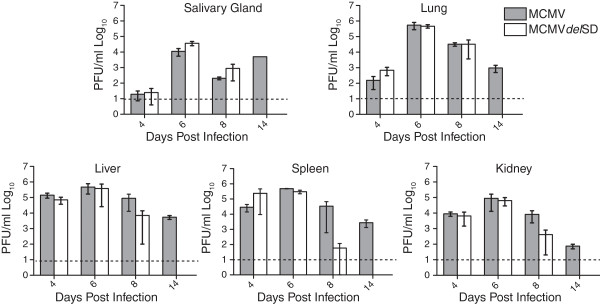
**Intron locus RNAs are dispensable for virus dissemination to the salivary glands during acute infection.** Three-month-old female BALB/c mice were inoculated i.p. with 5x10^5^ PFU of WT MCMV or MCMV*del*SD. At the indicated days post infection, organs were harvested from three mice per infection group to quantify infectious virus by plaque assay. Bars represent the mean and error bars represent the standard error of the mean (SEM). The dashed line indicates the limit of detection.

**Figure 8:**
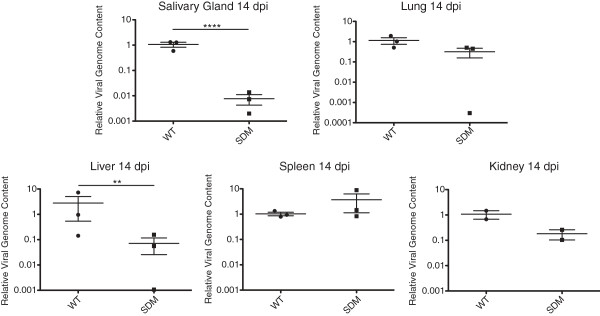
**Detection of viral genomes is significantly reduced in salivary glands during persistence.** Three-month-old female BALB/c mice were inoculated i.p. with 5x10^5^ PFU of WT MCMV (WT) or MCMV*del*SD (SDM). DNA was harvested from the indicated organs of three mice per infection group at 14 days post infection and viral genomes were quantified by qPCR using a primer probe set specific for the M54 MCMV gene and normalized to the beta-actin cellular gene. Bars represent the mean and error bars represent the standard error of the mean (SEM). *p* values represent the Student’s T Test result between WT MCMV and MCMV*del*SD infected mice (*p < 0.05 **p < 0.01 ***p < 0.001 ****p < 0.0001).

Although we have shown that recombinant viruses that fail to express the intron replicate poorly in the salivary glands, it was unclear if this attenuation was caused by a lack of dissemination to or a failure to replicate within the salivary gland [19]. To examine whether the intron is required for dissemination to the salivary gland, mice were inoculated with wild-type MCMV or MCMV*del*SD and viral yields in various tissues were measured at 4, 6, 8, and 14 days post infection (Figure 7). MCMV*del*SD replicated to similar levels as wild-type MCMV until 6 days post infection in all tissues examined. At 8 days post infection, levels of the splice donor mutant virus were significantly reduced in the liver, kidney, and spleen but were unchanged in comparison to wild-type MCMV within the salivary gland and lung. By 14 days post infection, replication of the splice donor mutant virus was severely attenuated in all organs assayed and infectious virus was below the limit of detection by plaque assay. Interestingly, the relative number of MCMV*del*SD genomes was reduced 100-fold in salivary glands at 14 days post infection relative to wild-type MCMV, suggesting that the virus was effectively cleared from this tissue (Figure 8). This data indicates that the intron does not influence viral dissemination to the salivary gland over a time course of infection but may function to promote viral persistence in the glandular epithelial tissue.

## Discussion

Non-coding RNAs are known to be expressed by nearly all herpesviruses that infect humans, yet the function of these RNAs in viral replication and pathogenesis has been elusive [15,31-34]. In our current study, we report further characterization of a lncRNA expressed by MCMV, the 7.2 kb RNA. This RNA is the ortholog of the 5.0 kb RNA of HCMV and we have previously shown that it is a virulence factor that promotes viral persistence *in vivo*. We showed that during productive infection in fibroblasts, the intron locus RNAs are transcribed with true late gene kinetics. These RNAs are derived from a common precursor RNA and are produced as the result of splicing of two exons that flank the intron to create a smaller mRNA and the 7.2 kb intron. We observed a doublet of bands with probes specific for the intron in northern blot analysis at 8.0 and 7.2 kb. While it is formally possible that these represent two different species of intron, we did not detect evidence of alternative splicing of a larger intron from the precursor RNA by sequencing of 5′-RACE products. We think it is likely that the intron remains in the form of a branched lariat after processing and therefore migrates more slowly during electrophoresis. Similar observations have been made for the LAT of HSV-1 [35]. We also detected a doublet of bands corresponding to the spliced mRNA in northern blot analysis. Again, sequencing of 5′-RACE products did not reveal any splicing variations that could account for the size differences. The polyadenylation chain lengths could differ for the individual spliced mRNA molecules representing the doublet observed for the mRNA. Identification of the transcriptional start sites rules out the possibility that a cluster of miRNAs mapped near to the 7.2 kb intron splice donor site originate from the same primary transcript [36]. It remains unknown, however, what functional relationship the miRNAs may have with the MCMV 7.2 kb intron locus, if any, during virus replication and pathogenesis.

Identification of the transcriptional start sites of the primary transcript expressed from the intron locus led us to identify putative transcriptional control elements that contribute to regulating transcription of the intron precursor. We identified two TATA box elements located within 135 bp of the TSS. However, using a luciferase reporter system, this region did not confer significant transcriptional activity above background. Transcriptional control elements driving expression of the adjacent M112/113 locus have been characterized and functioned in our assay as expected [30]. We were able to rule out the possibility that the M112/113 promoter controls transcription of the intron locus since function of this promoter is orientation dependent. Intron RNA and mRNA expression levels in cells infected with recombinant virus bearing a deletion spanning the distal and proximal TATA boxes and transcriptional start site were reduced compared to wild-type MCMV. However, the reduction in transcript levels was not nearly as robust as that observed in recombinants bearing mutations that affect intron stability or processing. We hypothesize that most of the variations we observed in the differing effects of promoter mutations on relative intron and mRNA levels can be accounted for by their different half-lives: since the intron is unusually stable, it appears to be less affected by the promoter deletions whereas the mRNA has a shorter half-life and the relative levels of RNA are more sensitive to modest reductions in transcriptional output associated with the promoter deletions.

Our studies did not identify sequence elements that robustly contribute to transcriptional control of this late transcriptional unit. As a late gene, amplification of genome copy number by DNA replication is thought to be critical for robust L transcription [2]. More recently, it has been demonstrated that viral replication and L gene expression also relies on a distinct set of five genes conserved across beta and gamma herpesviruses: UL79, UL87, UL91, UL92, and UL95 [37-39]. It is hypothesized that an RNA polymerase II transcriptional complex including one or more of these gene products is assembled to drive transcription of L genes. MCMV homologs of HCMV UL87, UL91, UL92, and UL95 have been annotated, but not tested for transcriptional activating functions [38-40]. M79, the MCMV homolog of HCMV UL79, has been shown to regulate L gene expression, although it does not appear to promote transcription of the intron locus [41]. It is also possible that we did not include the sequences responsible for binding of L gene transactivators within our reporter assay. Clearly, transcriptional regulation of L genes remains largely unexplored and it is likely that this process is significantly different from the activation of immediate early and early transcriptional units.

Post-transcriptional regulation, metabolism and function of lncRNAs is poorly understood in general. We demonstrated that the 7.2 kb intron accumulates as a consequence of a slow decay rate. Different properties contribute to lncRNA stability include GC content, the presence of specific decay elements, and if they are intronic. Given its intronic and AT-rich nature, the MCMV 7.2 kb lncRNA would be predicted to be highly unstable since introns are rapidly degraded on formation and AT-rich sequences do not form strong secondary structures that might protect the RNA from degradation. A stem-loop structure located near the 3′ end of the intron between the polypyrimidine track and putative branch point was identified using the structural prediction software mFold. Deletion of the stem loop does not impact processing of the precursor transcript, but does prevent accumulation of the intron during infection [19]. We hypothesize that the intron remains in the form of a lariat, similar to the Latency Associated Transcript (LAT) of HSV-1, thereby protecting it from degradation [42,43]. While the sequence and structural determinants of stability remain largely unknown for the 7.2 kb intron, its long half-life accounts for its accumulation and could be a key component of its functionality.

The spliced mRNA produced by processing of the 7.2 kb RNA spans the m106 ORF. Using an epitope-tagging strategy, we showed that this ORF could be translated during MCMV infection. The GFP-tagged protein co-localized to the nucleus of infected cells with the 7.2 kb RNA. This may reflect a related function of the intron and the m106 protein. Recombinant viruses that specifically disrupt m106 expression without impacting intron production will be useful reagents to probe the function for this viral protein. The m106 protein and its orthologues encoded by other CMVs, including UL106 of HCMV, have some unusual properties. Despite not sharing significant sequence homology, all UL106 orthologues are small (<150 amino acids), highly basic, arginine-rich peptides. It is unknown if other UL106 orthologues are expressed during infection, but given the conservation of the genomic organization of the intron locus among CMVs, it is a distinct possibility to be explored.

Production of the 7.2 kb RNA is required for the establishment of persistence in the salivary glands of mice. By analysis of multiple time points between the acute and persistent phases of infection in mice, we showed that recombinant virus lacking the intron appears to disseminate to the salivary gland as efficiently as wild-type MCMV. However, it is unable to maintain a highly productive replication program in the salivary glands as observed at 14 days post infection. In addition, we did not detect infectious MCMV*del*SD in any organs at 14 dpi and genome copy number of the mutant virus was substantially reduced in liver and kidney. It is possible that the mechanisms that prevent establishment of intron-mutant virus persistence in the salivary gland may also promote accelerated clearance of that virus from liver and kidney. At this time, the adaptive immune response acts to limit viral replication and it is possible that the 7.2 kb intron is involved in modulating immune surveillance in some way. Some cellular lncRNAs are involved in transcriptional regulatory processes, therefore, a possible mechanism for evading the immune response could be to regulate cellular or viral genes that are involved in this host pathogen relationship [44,45].

## Conclusions

This current study has provided a detailed transcriptional and functional analysis of the MCMV 7.2 kb RNA locus. Mapping the topography of the 7.2 kb RNA locus allows us to understand the genomic elements that not only comprise the locus but also control its expression. The unusual stability of the 7.2 kb RNA compensates for the weak transcriptional output by the putative promoter region observed and may also provide insight towards molecular function within the nucleus of infected cells. Uncovering the stability determinants of the 7.2 kb RNA will therefore allow us to understand the mechanisms that promote its retention within infected cells. Although a function has yet to be determined to the 7.2 kb RNA, this current analysis has provided the framework for investigating its function during viral persistence.

## Methods

### Cell culture

10.1-mouse embryonic fibroblasts were propagated in Dulbecco’s modified Eagle’s medium (DMEM) supplemented with 10% newborn calf serum, 100 U/ml penicillin, and 100 ug/ml streptomycin. Cells were maintained at 37°C with 5% CO_2_.

### Viruses

The BAC clone of the wild type Smith MCMV strain, pSM3fr, was used as the parent strain in this study [46,47]. Recombinant viruses were generated by linear recombination using either the seamless, red-mediated recombination in the DH10B *Escherichia coli* strain GS1783 method or by using the FLP-recombinase method as previously described [19]. Primer sequences used to generate the recombinant viruses are indicated in Table 2. Recombinant BAC DNA was isolated and electroporated into 10.1 fibroblasts to produce viral stocks. Multi-step growth analysis was performed by infecting 10.1 fibroblasts at a multiplicity of infection (MOI) of 0.05, collecting supernatant every 24 hours for five days starting at time zero, and titrating the supernatant by plaque assay or TCID_50_ assay on 10.1 fibroblasts.

**Table 2:** Primers used for recombinant virus production

**Target**	**Sequence 5′ to 3′**
MCMV*del*20	GGAGTGTAGGTATTCACCGTCAGACGCAACCTGACGCATCCCGGCTAGAATCGATTTATTCAACAAAGCCACG
	CACCTGAGCCTGCTCGGCCGTTCGCTCAGGTGTGATAATGCACCTTTCAGCGCGTATATCTGGCCCGTACATCG
	TATTCACCGTCAGACGCAACCTGACGCATCCCGGCTAGAACTGAAAGGTGCATTATCACACCTGAGCGAACGGCCGAGCA
	TGCTCGGCCGTTCGCTCAGGTGTGATAATGCACCTTTCAGTTCTAGCCGGGATGCGTCAGGTTGCGTCTGACGGTGAATA
MCMV*del* 100	GATCACGCTACCACCGTGTGTCTCCGTACTCCGCTATTATACTTTGCGGCTCGATTTATTCAACAAAGCCACG
	CGCTACCACCGTGTGTCTCCGTACTCCGCTATTATACTTTGCGGCCTGAAAGGTGCATTATCACACCTGAGCGAACGGCCGAGCAGGCTC
	GAGCCTGCTCGGCGTTCGCTCAGGTGTGATAATGCACCTTTCAGGCCGCAAAGTATAATAGCGGAGTACGGAGACACACGGTGGTAGCG
MCMV*del* 135	CGGCACGGGGAAATAAAATGATCACGCTACCACCGTGTGTCTCCGTACTCTCGATTTATTCAACAAAGCCACG
	GATGCGTCCGCCGCCTCACCTGAGCCTGCTCGGCCGTTCGCTCAGGTGTGCGCGTATATCTGGCCCGTACATCG
	AAATAAAATGATCACGCTACCACCGTGTGTCTCCGTACTCCACACCTGAGCGAACGGCCGAGCAGGCTCAGGTGAGGCGG
	CCGCCTCACCTGAGCCTGCTCGGCCGTTCGCTCAGGTGTGGAGTACGGAGACACACGGTGGTAGCGTGATCATTTTATTT
M106-GFP	TCCACCAACACGATCCCCGAGATACCCAGAATCGTGGTCGAGGTGGTAGACGCCGGAAGAAGATGGAAAAAG
	GTTTTCTGACATGAGTCTGTGTGTTTATTTATTAATTATCTGTCAGTTTACGTCGTGGAATGCCTTCG

### Plasmids

RACE products were cloned into pGEM-T-Easy (Promega) and sequenced. Reporter constructs were generated by PCR amplifying sequence upstream of the 7.2 kb RNA splice donor site. Primers used to generate the reporter constructs are indicated in Table 1. Amplicons were resolved by gel electrophoresis and gel purified using the Qiaquick Gel Extraction Kit (Qiagen). Amplicons were cloned into pGEM-T-Easy. After insertion into the pGEM-T-Easy plasmid, the inserts were digested from the plasmid using the flanking EcoRI sequences then subcloned into the pGL3 Basic vector at a newly generated EcoRI site using the site directed mutatgenesis kit (Stratagene). Orientation of the cloned insert was determined by sequencing the pGL3 plasmid. The pGL3-SV40 control plasmid and the renilla phRL-TK normalization control plasmid were used in the luciferase assays (Promega).

### Promoter analysis

Mouse fibroblasts were seeded in 24-well dishes and co-transfected with a pGL3 construct (see above) and phRL-TK using polyethylenimine (PEI) at a 6:1 ratio of PEI to plasmid DNA. The plasmids were cotransfected at a 1:1 ratio with 3 × 10^10^ copies per well of each plasmid. Protein lysates were harvested 48 hours post transfection and assayed following the Duo-Glow Luciferase assay kit (Promega, Madison, WI). Luciferase activity was normalized to renilla activity in each well and the data is expressed as the fold change of luciferase induction relative to the luciferase induction from the pGL3 promoter-less vector.

### RNA analyses

To determine expression kinetics, 10.1 fibroblasts were pretreated with 100 μg/mL cyclohexamide or 200 μg/mL PAA 1 hour before MCMV infection. Total RNA was harvested from 10.1 fibroblasts at either 24 hours post infection (h p.i.) or 48 h p.i. with TRIzol LS (Life Technologies) according to the manufacturer’s protocol. RNA was resolved on either a 1.4% or 0.7% glyoxal gel for detection of the spliced mRNA or 7.2 kb RNA respectively. Northern blot analysis for intron locus RNAs was carried out as previously described using specific radio-labeled riboprobes [14].

RNA half-life analysis was performed by infecting fibroblasts with wild-type MCMV at an MOI of 1.0. At 30 hours post infection, 4 ug/mL Actinomycin D was added to infected cells and RNA was harvested over a time course starting at time 0 and ending at 32 hours post treatment. Transcript levels were quantified by qRT-PCR at the different times points relative to RNA at time 0.

### 5′ and 3′ RACE

Total RNA harvested from mock or WT MCMV infected 10.1 mouse fibroblasts at 48 hours post infection was analyzed by the First Choice RNA ligase-mediated rapid amplification of cDNA ends kit as recommended by the manufacturer (RLM-RACE, Ambion). Amplification products were purified and TA cloned into pGEM-T-Easy (Promega, Madison, WI) and sequenced (Table 1). For 3′ RACE, RNA was reverse transcribed using a poly(A)-adapter. Amplification products were cloned and sequenced. MacVector software was used to align RACE sequences to MCMV reference sequence.

### Primer extension

Oligonucleotides 497 and 50 were end radiolabeled and used in primer extension reactions on total RNA from mock- or MCMV-infected cells as previously described (Table 1) [19]. Primer extension products were analyzed by denaturing 10% urea-polyacrylamide gel electrophoresis followed by phosphorimager analysis.

### qRT-PCR and qPCR

Total RNA was DNase treated and reverse transcribed using the Quantitect Reverse Transcription kit (Qiagen). Quantitative PCR was performed using the LightCycler 480 Probes Master Mix (Roche) along with IDT hydrolysis probes specific for the intron locus RNAs and selected housekeeping genes (Table 1). Ct values were determined using the Basic Relative Quantification analysis module of the LightCycler 480 (Roche) software. Primer-probe efficiencies were determined by three biological replicates of 10-fold dilutions. The 18S rRNA was used as a reference gene and the relative target levels were quantified by a delta-delta CT method, the Pfaffl method, that incorporates the calculated primer-probe efficiencies (Table 1) [48].

### Immunoblotting

Cells were trypsinized, centrifuged, and collected in PBS. Cells were lysed in RIPA buffer (150 mM NaCl, 1% v/v Nonidet P-40, 0.5% w/v deoxycholate, 0.1% w/v SDS, 5 mM EDTA, 50 mM Tris; pH 8.0) containing protease inhibitor cocktail (Roche). The cell lysate was briefly sonicated to facilitate nuclear protein release and insoluble debris was centrifuged. GFP tagged m106 protein was immunoblotted using a rabbit polyclonal antibody and detected with a fluorescently conjugated secondary antibody using the SuperSignal West Pico Chemiluminescent Substrate (Thermo Scientific). HP1, heterochromatin associated protein 1, was detected similarly as a loading control (Santa Cruz).

### FISH and Immunofluorescence

Fluorescently labeled RNA probes antisense to the 7.2 kb intron were generated using the FISH Tag kit (Table 1) (Invitrogen). Briefly, probes were in vitro transcribed from linearized pGEM-T-Easy constructs using an amino allyl modified base in which an alexa flour can be chemically attached to. Following in vitro transcription of the probes, the DNA templates are digested using DNase I and the amino modified RNA is purified over a column then ethanol precipitated. The purified probes are fluorescently labeled according to the manufacturer’s instructions then column purified and subsequent ethanol precipitation. Cells were fixed for 20 minutes in 4% paraformaldehyde, 10% acetic acid in 1x PBS. The fixation was quenched for 20 minutes in PBS with 0.1 M glycine. Cells were washed twice with PBS then permeabilized with 70% ethanol overnight at 4°C. Cells were rehydrated by washing twice with 50% formamide/2x SSC. The probe was denatured by heating at 65°C for 10 minutes in probe buffer then cells were incubated overnight with the denatured probe at 37°C. The following day, cells were washed twice with 0.1X SSC/50% formamide at 50°C then washed once with PBST. Immunoflourescence of m106-GFP was carried out as previously described [49].

### Mice

All animal procedures were approved by the Institutional Animal Care and Use Committee of the University of Colorado Denver. BALB/c mice were inoculated by intraperitoneal injection with 5×10^6^ pfu of tissue culture derived wild type or recombinant MCMV in 300 ul DMEM. At designated times mice were sacrificed and liver, spleen, lungs, kidneys, and salivary glands were removed and weighed. Part of the tissue was homogenized and titrated on mouse fibroblasts. The remaining tissue was processed for DNA isolation in order to quantify viral genomes using the DNeasy Blood and Tissue kit according to the manufacturer’s protocol (Qiagen). 250 ng of DNA was analyzed by qPCR for each sample.

## Competing interests

The authors declare that they have no competing interests.

## Author contributions

TMS, L-AMV, CGA, and CAK participated in the design and execution of the study. TMS and CAK drafted the manuscript. All authors read and approved the final manuscript.
